# A flea-associated Rickettsia pathogenic for humans.

**DOI:** 10.3201/eid0701.010112

**Published:** 2001

**Authors:** D Raoult, B La Scola, M Enea, P E Fournier, V Roux, F Fenollar, M A Galvao, X de Lamballerie

**Affiliations:** Unité des Rickettsies, CNRS UPRESA 6020, Faculté de Médecine, Université de la Méditerranée, 27 Bd Jean Moulin, 13385 Marseille, France. Didier.Raoult@medecine.univ-mrs.fr

## Abstract

A rickettsia named the ELB agent, or "Rickettsia felis," was identified by molecular biology techniques in American fleas in 1990 and later in four patients from Texas and Mexico. We attempted to isolate this rickettsia from infected fleas at various temperatures and conditions. A representative isolate of the ELB agent, the Marseille strain, was characterized and used to develop a microimmunofluorescence test that detected reactive antibodies in human sera. The ELB agent was isolated from 19 of 20 groups of polymerase chain reaction-proven infected fleas. The microimmunofluorescence results provided serologic evidence of infection by the ELB agent in four patients with fever and rash in France (2) and Brazil (2), supporting the pathogenic role of this rickettsia. Our successful isolation of this rickettsia makes it available for use in serologic tests to determine its clinical spectrum, prevalence, and distribution.


### A Flea-Associated Rickettsia Pathogenic for Humans

**Didier Raoult,* Bernard La Scola,* Maryse Enea,***
**Pierre-Edouard Fournier,* Véronique Roux,***
**Florence Fenollar,* Marcio A.M. Galvao,† Xavier de Lamballerie***
* Unité des Rickettsies, CNRS UPRESA 6020, France;
†Ouro Preto Federal University, Brazil

A rickettsia named the ELB agent, or "*Rickettsia felis*," was identified by molecular biology techniques in American fleas in 1990 and later in four patients from Texas and Mexico. We attempted to isolate this rickettsia from infected fleas at various temperatures and conditions. A representative isolate of the ELB agent, the Marseille strain, was characterized and used to develop a microimmunofluorescence test that detected reactive antibodies in human sera. The ELB agent was isolated from 19 of 20 groups of polymerase chain reaction-proven infected fleas. The microimmunofluorescence results provided serologic evidence of infection by the ELB agent in four patients with fever and rash in France (2) and Brazil (2), supporting the pathogenic role of this rickettsia. Our successful isolation of this rickettsia makes it available for use in serologic tests to determine its clinical spectrum, prevalence, and distribution.

*Rickettsia* are intracellular Proteobacteria associated with arthropods, including body lice, fleas, ticks, and mites (1). *R. typhi*, the agent of murine typhus, is transmitted by rat fleas, *Xenopsylla cheopsis*. In 1990, when cat fleas (*Ctenocephalides felis*) were examined as possible vectors of *R. typhi*, a novel *Rickettsia*-like organism was observed by electron microscopy in midgut epithelial cells of the fleas. The agent, named the ELB agent for the EL Laboratory (Soquel, CA) (2), was detected in 1994 and 2000 by polymerase chain reaction (PCR) in four patients from Texas and Mexico (3,4). The taxonomic position of this organism within the genus *Rickettsia* was assessed by genomic sequence comparison, following the successful amplification of a 17-kDa protein gene fragment from infected flea tissue by PCR with genus-specific primers (5). In addition, the organism was found to be transmitted transovarially in fleas (5) and to be pathogenic in a human patient (6). In 1995, the name "*R. felis*" was proposed for the ELB agent on the basis of its phenotypic characteristics, as well as its clear genotypic differences from other known *Rickettsia* species (6). The organism was provisionally named "*R. felis*" but the name is not formally approved by International Society for Systematic and Evolutionary Biology as no strain was deposited in any official collection. In 1997, the ELB agent was detected in two other flea species in the United States, *C. felis* and *Pulex irritans* (7). Although isolation in tissue culture was reported (3,8,9), contamination with *R. typhi* has hampered subsequent work (6), so no isolate of *R. felis* was available when we began our study.

Address for correspondence: D. Raoult, Unité des Rickettsies, CNRS UPRESA 6020, Faculté de Médecine, Université de la Méditerranée, 27 Bd Jean Moulin, 13385 Marseille Cedex 05, France; fax: 33-4-91-38-7772; e-mail: Didier.Raoult@medecine.univ-mrs.fr.

We describe methods used to cultivate several isolates of the ELB agent and its morphologic, antigenic, and genomic characteristics, as well as the results of a serosurvey with one of our type strains.

### Materials and Methods

*C. felis* fleas (Flea Data Inc., Freeville, NY) were divided into 20 groups of 5. After surface sterilization by a 5-minute immersion in 70% methanol with 0.2% iodine, the fleas were washed in sterile distilled water and frozen in liquid nitrogen. Frozen fleas were macerated with a sterile plastic spatula, suspended in 0.8 mL of culture medium, and injected into shell vials. Fifty microliters of the suspension was retained for use as template in a *Rickettsia*-specific PCR targeting a fragment of the citrate synthase-encoding gene (*glt*A) (10).

Human embryonic lung fibroblasts (11) or the XTC-2 cell line derived from *Xenopus laevis* (12) were used for isolation by the shell vial centrifugation technique (6). Subconfluent cell monolayers were obtained by incubating the shell vials at 28°C for 48 hours after they were injected with 50,000 cells in 1 mL of Leibowitz-15 medium with L-glutamine and L-amino-acids (GIBCO, Rockville, MD), 5% (v/v) fetal calf serum and 2% (v/v) tryptose phosphate (GIBCO) for XTC-2 cells, and Minimum Essential Medium (GIBCO) supplemented with 2 mM L-glutamine and 10% fetal bovine serum for human embryonic lung fibroblasts. Before injection with the flea extract, the medium was removed by aspiration.

After injection of both cell types with suspensions of five fleas resuspended in 0.8 mL of the corresponding medium, the shell vials were centrifuged at 700 X *g* for 1 hour at 20°C, and the supernatant was discarded. After two washings in sterile phosphate-buffered saline (PBS), 1 mL of fresh medium containing 4 µg/mL cotrimoxazole, an antibiotic used to prevent contamination, was added to the shell vials, which were incubated at 28°C. The cell culture medium was replaced every 7 days for up to 30 days. When rickettsiae could be detected in cells in the discarded medium by Gimenez staining (13), the infected cell monolayer was harvested and spread onto a subconfluent cell monolayer of the same cell line in a 25-$cm^2$ tissue culture flask, which was also incubated at 28°C.

For further studies, we used our first isolate of the ELB agent, which we named the Marseille strain. We attempted to infect mammalian cell lines with the Marseille isolate on monolayers of Vero, MRC-5, and L-929 cells incubated with 5% $CO_2$ at 28°C, 32°C, or 37°C, in the same medium as human embryonic lung fibroblasts. The ultrastructure of the Marseille isolate was studied by electron microscopy (14), and the strain was purified as for other rickettsia (15).

To quantify growth of the ELB agent, 1 µL of $10^{-1}$, $10^{-2}$, $10^{-3}$, $10^{-4}$, $10^{-5}$, $10^{-6}$ dilutions of a suspension made of rickettsiae harvested from a 25-$cm^2$ tissue culture flask and purified from cells were deposited onto 30-well microscope slides (Dynatech Laboratories Ltd., Billingshurt, UK), air dried, fixed with acetone for 10 minutes, and then stained by the Gimenez technique. The number of rickettsiae was estimated visually.

To raise polyclonal antisera against the Marseille isolate, 6- to 8-week-old Balb C mice were injected intraperitoneally with 1 mL of a solution containing v/v $10^6$ purified Marseille isolate in PBS and complete Freund's adjuvant. Inoculation was repeated at 10, 20, and 30 days. At 40 days, blood was collected by intracardiac puncture, and sera were stored at –20°C. The same procedure was performed with *R. conorii* (Moroccan strain, ATCC VR 141) and *R. typhi* (Wilmington strain, ATCC VR-144).

To determine the prevalence of antibodies reactive with the organism in the general population in France, serum specimens from 100 French blood donors were tested by microimmunofluorescence against the Marseille strain; 140 serum samples from Brazilian blood donors, which had been sent to our laboratory to estimate the seroprevalence of various rickettsioses, were also tested. Microimmunofluorescence was also used to determine cross-reactivity between our strain of the ELB agent and other rickettsiae. Convalescent-phase serum specimens from 67 patients with epidemiologic, clinical, and serologic evidence of epidemic typhus, 16 patients with murine typhus, and 97 French patients with fever and rash serologically and clinically diagnosed as Mediterranean spotted fever, were tested by microimmunofluorescence for antibodies against *R. rickettsii, R. typhi,* and the Marseille strain. Serum samples from 16 Brazilian patients with unexplained febrile rash were also included in these tests. For microimmunofluorescence, *R. conorii* strain Seven (Malish)*, R. rickettsii* strain R (Bitterroot)*, R. prowazekii* strain Brein L, and *R. typhi* strain Wilmington were grown in Vero cells and purified (11). These antigens and the purified suspension of the Marseille strain described above were applied at separate sites on each well of 30-well microscope slides (Dynatech Laboratories, Ltd.), air dried, and fixed with acetone for 10 min. Microimmunofluorescence tests were performed (16), with immunoglobulin (Ig) G and IgM titers determined separately. To remove antibodies against any host-cell components, antisera were absorbed with XTC-2 or Vero cells before being used in the microimmunofluorescence. Moreover, before detection of IgM, the antisera were absorbed with rheumatoid factor absorbent (Behring-Werke AG, Marburg, Germany). The antisera were then applied to the fixed antigens at doubling dilutions from 1:4 to 1:2,048 (16).

Analysis of major proteins of the Marseille isolate by sodium dodecyl-sulfate polyacrylamide gel electrophoresis (SDS-PAGE) were performed by using both heated and unheated antigens (17). Major immunogenic proteins were studied by Western blot with purified unheated antigens (14,17).

For PCR amplification and sequencing of gene fragments from macerated flea suspensions and rickettsial isolates, genomic DNA was extracted by using the QIAmp tissue kit (Qiagen, Hilden, Germany) according to the manufacturer's instructions. In addition to the 17-kDa antigen- and citrate synthase-encoding genes determined by Azad et al. (5) and Higgins et al., respectively (3), we amplified and sequenced fragments of the genes encoding the rickettsial outer membrane proteins A and B (*omp*A and *omp*B). Primers were designed within conserved regions of the genes, and amplifications were carried out as described (Table 1,18). Sequencing reactions with these primers were done with the dRhodamine Terminator sequencing kit (PE Applied Biosystems, Warrington, UK). The reaction products were resolved on 5% polyacrylamide gels (Tebu, Le Perray en Yvelines, France) by an ABI PRISM 377 DNA sequencer (PE Applied Biosystems). Amplifications of *R. typhi* by PCR from subcultures of the Marseille strain were performed by using the *glt*A-derived *R. typhi*-specific primers TY1f (5'-TGGGGAACTACCAAGTAGT-3') and TY1r (5'-ACCAGTGCTAATACATGCAA-3') as described to determine the purity of the culture. DNA was extracted as described. DNA from *R. typhi* cultured in Vero cells was used as positive control.

Nucleotide sequences were aligned with sequences from other *Rickettsia* species in GenBank by using the multisequence alignment software CLUSTAL within the BISANCE environment (20). The phylogenetic relationships between our strain and other representative

Table 1. Primers used for PCR amplification and sequencing of the ELB agent strain Marseille-URRWFXCal$_2$

| Gene | Primer | Nucleotide sequence (5'-3') | Gene position relative to open reading frame | Reference |
|---|---|---|---|---|
| 17-kDa antigen | 17kDF[a] | GCTCTTGCAACTTCTATGTT | 31-50 | This manuscript |
| | 17KdR[a] | CATTGTTCGTCAGGTTGGCG | 464-445 | This manuscript |
| Citrate synthase | CSFEL1[a] | TGATTCAGAATTTGCCGAAT | 21-40 | 17 |
| | CS890r[a] | GCTTTAGCTACATATTTAGG | 890-871 | 17 |
| | CS532r[a] | GCCGCAATGTCTTATAAATATTCT | 532-555 | This manuscript |
| | CS1273r[a] | CATAACCAGTGTAAAGCTG | 1273-1255 | This manuscript |
| | CS244r[b] | CTTTAATATCATATCCTCGAT | 244-224 | 17 |
| | Rp877P[b] | GGGGACCTGCTCACGGCGG | 797-815 | 18 |
| rOmpB | 120-M59[a] | CCGCAGGGTTGGTAACTGC | M59-M41 | This manuscript |
| | 120-807[a] | CCTTTTAGATTACCGCCTAA | 807-788 | This manuscript |
| | 120-607[a] | AATATCGGTGACGGTCAAGG | 607-626 | This manuscript |
| | 120-1497[a] | CTATATCGCCGGTAATT | 1497-1480 | This manuscript |
| | 120F-1351[a] | TTTAGGAAACGCTGGTTCTC | 1351-1370 | This manuscript |
| | 120F-2934[a] | GCGTTAGTTGCGATAATACT | 2934-2915 | This manuscript |
| | 120-2788[a] | AAACAATAATCAAGGTACTGT | 2788-2808 | This manuscript |
| | 120-3599[a] | TACTTCCGGTTACAGCAAAGT | 3599-3579 | This manuscript |
| | 120F-3440[a] | GTTAATGCAACAACTACGGG | 3440-3459 | This manuscript |
| | 120F-4341[a] | GCATCGAAGAAGTAACGCTG | 4341-4322 | This manuscript |
| | 120-4232[a] | GGTTTCTCATTCTCTCTATATGG | 4232-4254 | This manuscript |
| | 120-4879[a] | TTAGAAGTTTACACGGACTTT | 4879-4857 | This manuscript |
| | 120F-1749[b] | CTATTAGTGGTAATATTGGTAC | 1749-1770 | This manuscript |
| | 120F-2495[b] | CATGGTCATTACCTGCATTACC | 2495-2481 | This manuscript |
| | 120-4489[b] | GTCTTCTGACGAAAACTACAA | 4489-4509 | This manuscript |
| ROmpA | 190-70[a] | ATGGCGAATATTTCTCCAAAA | 70-90 | 18 |
| | 190-701[a] | GTTCCGTTAATGGCAGCATCT | 701-681 | 19 |

[a]Primers used for both PCR amplification and sequencing of the ELB agent strain Marseille-URRWFXCal$_2$.
[b]Primers used only for sequencing.

strains based on the analysis of *glt*A and *omp*B sequences were determined by using the Phylip software (21). The distance matrix generated by DNADIST was determined under the assumption of Jukes and Cantor (22) and used to construct a dendrogram by the neighbor-joining method (23). Two other dendrograms were constructed by using data processing with the maximum-likelihood and parsimony program DNAPARS. Bootstrap replicates were performed by using SEQBOOT and CONSENSE in the PHYLIP software to estimate the node reliability of the trees obtained by the three methods (24).

To improve the sensitivity of PCR and detect rickettsial DNA from serum samples, we designed a nested PCR based on the amplification of the *glt*A gene. DNA was extracted from 200 µL of serum by using the QIAmp blood kit (Qiagen) as recommended by the manufacturer. External primers designed for this purpose were ELB1f (5'-CTGCTTCTTGTCAGTCTAC-3') and ELB1r (5'-GATTTTTTGTTCAGGGTCTTC-3'), and internal primers were ELB2f (5'-GGAATCT TGCGACATCGA-3') and ELB2r (5'-CAGCCTACG GTTCTTGC-3'). The internal primers encompassed a 952-bp *glt*A fragment allowing a reliable identification of most rickettsial species after sequencing (including *R. felis*, *R. conorii*, and *R. rickettsii*). Amplification, sequencing of amplicons, and sequence analysis were done as described. Forty-seven serum samples were tested with this technique, 27 from patients with rickettsial diseases (including those reacting serologically to the ELB agent) and 20 from blood donors used as negative controls.

### Results

The ELB agent was detected by PCR amplification of a *glt*A fragment from all the flea suspensions used to inoculate the cell culture monolayers. Initial attempts to isolate the rickettsia in human embryonic lung fibroblasts failed, but growth was observed by Gimenez staining and the rickettsiae were confirmed as the ELB agent by PCR analysis: 100% homology was observed with DNA amplified with the *gltA* sequence of the ELB agent in 19 of 20 supernatants from the XTC-2 cell monolayers grown at 28°C 7 and 14 days after inoculation. *R. typhi* DNA could not be detected by PCR in any of the cell culture supernatants, whereas the primer pair Ty1f and TY1r amplified positive control DNA.

Initial isolation required 14 days, and subsequent passages required 6 days to detect the ELB agent. Cultures of all isolates were easily established in XTC-2 cells from the 19 suspensions. The reference strain, Marseille-URRWFXCal$_2$, has been deposited (accession number I-2363) in the French National Culture Collection (Institut Pasteur, Paris, France) and will be sent to the American Type Culture Collection. Before further studies of this strain, we reconfirmed by PCR that our strain of the ELB agent was not contaminated with *R. typhi*.

The Marseille strain grew most rapidly at 28°C in XTC-2 cells, which died at temperatures ≥32°C (Table 2). Human embryonic lung fibroblasts do not multiply at 28°C, as their optimal growth temperature is 37°C, and therefore the ELB agent could not be cultivated in this cell line. The organism also grew in Vero cells incubated at either 28°C or 32°C but at half the rate of growth observed in XTC-2 cells. The MRC-5 and L-929 cell lines were unable to support permanent growth of the Marseille strain. Electron microscopy showed the rickettsia to be present and free in the cytoplasm but not in the nucleus of the cells (Figure 1).

Table 2. Time required to infect 90% of cells in a 174-cm$^2$ cell culture flask after inoculation with 5 x10$^5$ ELB agent strain Marseille-URRWFXCal$_2$

| | Incubation temperatures | | |
|---|---|---|---|
| Cell type | 28°C | 32°C | 37°C |
| XTC-2 cells | 6 days | ND[a] | ND[a] |
| Vero cells | 14 days | 14 days | No |
| MRC-5 cells | NG[b] | NG[b] | NG[b] |
| L-929 cells | NG[b] | NG[b] | NG[b] |

[a]ND = not done. XTC-2 cells died at temperatures 32°C.
[b]NG = no growth was obtained

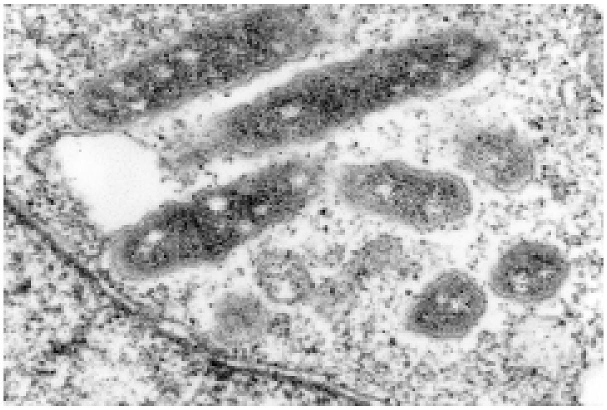

Figure 1. Transmission electron micrograph of the ELB agent in XTC-2 cells. The rickettsia are free in the cytoplasm and surrounded by an electron transparent halo. Original magnification X 30,000.

Four gene fragments were successfully amplified by PCR, and the base sequences of both DNA strands of each segment were determined twice. Nonambiguous sequence data were obtained between bases 51 and 444 of the 17-kDa antigen-encoding gene (394 base pairs), bases 41 and 1236 of *gltA* (1196 bp), bases 91 to 665 of *ompA* (575 bp), and bases 1 to 1236 of *ompB* (1236 bp). Sequences of the 17-kDa protein-encoding gene and *gltA* were 100% homologous with those in GenBank (accession numbers M82878 and U33922, respectively). The GenBank accession numbers for the Marseille strain of the ELB agent nucleotide sequence data reported in this paper are as follows: citrate synthase-encoding gene, AF210692; 17-kDa protein-encoding gene, AF210693; outer membrane protein A-encoding gene, AF210694; and outer membrane protein B-encoding gene, AF2106695. Phylogenetic analysis inferred from the comparison of *gltA* and *ompB* nucleotide sequences with the three analysis methods produced similar organizations. The ELB agent clustered with *R. akari* and *R. australis* (Figure 2).

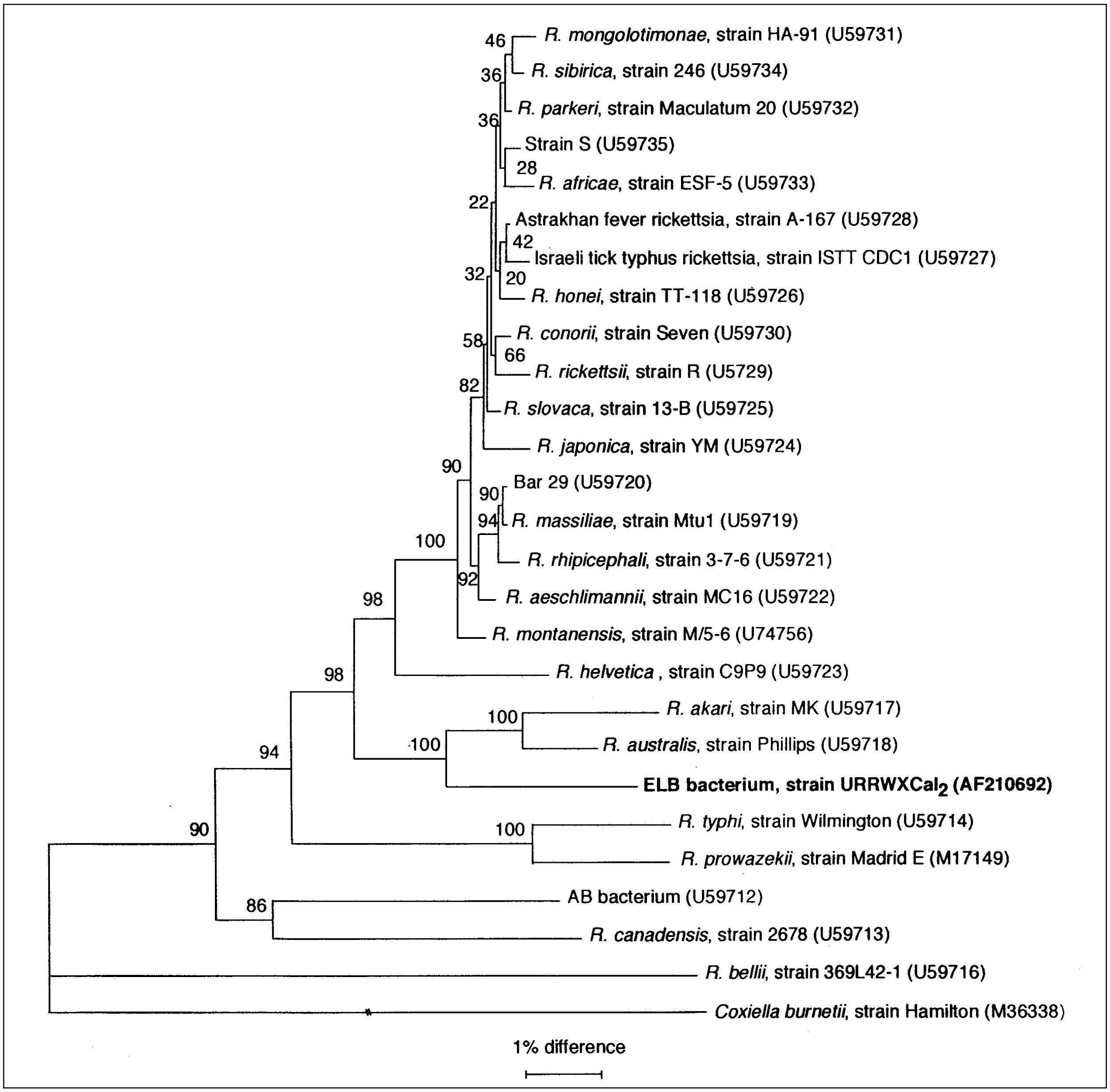

Figure 2. Phylogenetic tree of members of the genus *Rickettsia* inferred from comparison of *gltA* sequences by using the neighbor-joining method. Bootstrap values for the nodes are indicated.

The SDS-PAGE profile of the ELB agent differs from those of *R. conorii* and *R. typhi* (Figure 3). The ELB agent had a high molecular weight protein with a molecular mass of >150 kDa, which was not present in *R. typhi* (Figure 3), as well as a 30-kDa heat-labile protein not present in *R. typhi* or *R. conorii.* Mouse antisera had a 1:1,600 IgG titer against the ELB agent. In Western blots (Figure 3), mouse antisera to the ELB agent reacted strongly with high molecular mass proteins of the agent, another protein of about 30 kDa, and the lipopolysaccharide antigens. Mouse antisera cross-reacted weakly with the high molecular-mass protein antigens of *R. typhi* and *R. conorii*.

None of the serum specimens from the French or Brazilian blood donors had substantial antibody titers against the ELB agent. Of the 67 sera from patients with epidemic typhus, 51 (76%) had antibodies reactive with both *R. prowazekii* and the ELB agent; 66 of these sera had lower titers to the ELB agent than to *R. prowazekii*, and one specimen had the same titer to both organisms. Eleven of the sera from the 16 patients with murine typhus contained antibodies reactive with both *R. typhi* and the ELB agent, but titers were lower against the ELB agent in each specimen, except for one with identical titers to the ELB agent and *R. typhi*. Of the 97 sera from patients with suspected Mediterranean spotted fever, 30 had antibodies to *R. conorii* only, and 67 had antibodies reactive with both *R. conorii* and the ELB agent, with titers identical in one patient and greater against the ELB agent in two patients (patients 1 and 2, Table 3). These two patients, a woman and a man from Marseille, had febrile exanthema in 1995 and 1998, respectively. The other 64 patients had higher antibodies to *R. conorii* than to the ELB agent. Among the Brazilian patients with febrile rash, nine had higher antibody levels to *R. rickettsii*, the agent of Rocky Mountain spotted fever, and the ELB agent; one had higher titers to *R. typhi*; and two had higher titers to the ELB agent (patients 3 and 4, Table 3). These two patients had fever, rash, vomiting, and stupor. Patient 3, in whom the ELB agent was identified by sequencing following nested-PCR amplification

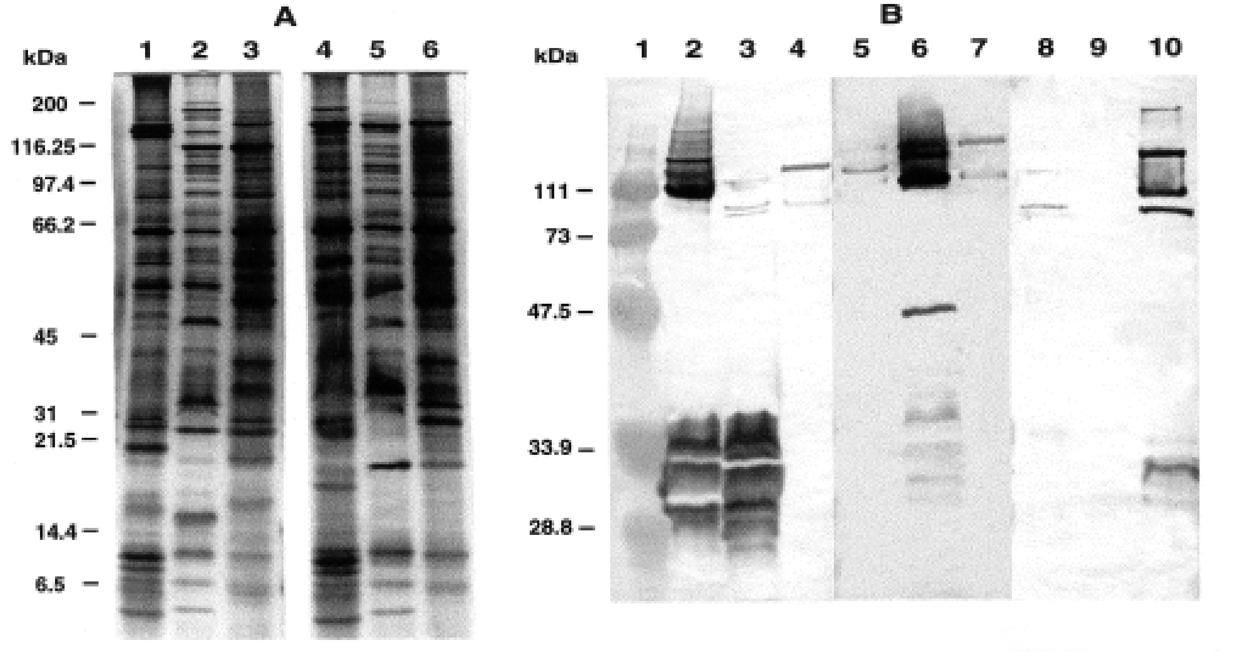

Figure 3. (A) Silver-stained SDS-PAGE of whole-cell protein preparations of *Rickettsia conorii*, the ELB agent and *R. typhi*. Lane 1, *R. conorii*; lane 2. ELB agent; lane 3, *R. typhi*; lane 4, heated *R. conorii*; lane 5, heated ELB agent; lane 6, heated *R. typhi*. Molecular weights are indicated on the left. (B) Western blot of rickettsial proteins probed with various antisera. *R. conorii* antigens (lanes 2, 6, and 10), ELB agent antigens (lanes 3, 7, and 11) and *R. typhi* antigens (lanes 4, 8, and 12) were probed with anti-*R. conorii* (lanes 2 to 4), anti-ELB agent (lanes 5 to 7) and anti-*R. typhi* (lanes 8 to 10) polyvalent mouse antisera. Lane 1: molecular weight marker.

Table 3. Serologic studies of French (patient 1 and 2) and Brazilian (patient 3 and 4) patients with serologic evidence of infection with the ELB agent

| Patient | Antibody titers to ELB agent | | *Rickettsia prowazekii* | | *R. typhi* | | *R. conorii* | | *R. rickettsii* | |
|---|---|---|---|---|---|---|---|---|---|---|
| no. | IgG | IgM | IgG | IgM | IgG | IgM | IgG | IgM | IgG | IgM |
| 1 | 2,048 | 64 | 0 | 0 | 0 | 0 | 64 | 64 | ND[a] | ND |
| 2 | 512 | 32 | 0 | 0 | 0 | 0 | 128 | 32 | ND | ND |
| 3 | 1,024 | 64 | ND | ND | 0 | 64 | ND | ND | 64 | 64 |
| 4 | 512 | 64 | ND | ND | 0 | 64 | ND | ND | 128 | 64 |

[a]ND: not done from a serum sample, had coma, thrombocytopenia 71,000/mL, and aspartate aminotransferase transaminase 75 IU/mL. Three additional Brazilian patients had similar titers against *R. rickettsii* and the ELB agent. Overall, four patients were identified as infected by the ELB agent, and five had equally elevated antibody levels to the ELB agent and another *Rickettsia*.

Of the 47 serum samples tested, only two were positive by the nested PCR. In addition to detecting the ELB agent in patient 3, this technique also identified *R. rickettsii* in a Brazilian patient who had much higher titers to *R. rickettsii* than to the ELB agent. None of the 20 negative controls reacted in the assay.

### Conclusions

Several arthropod-borne pathogenic viruses and bacteria grow more rapidly in the laboratory at temperatures lower than human body temperature (25,26). *Rickettsia* are also better adapted for growth at low temperatures. One of the main microbiologic differences between the typhus and spotted fever groups is their optimal growth temperatures. While members of both groups can be maintained in embryonated eggs at 37°C, optimal growth of the typhus group of rickettsiae is 35°C and of the spotted fever group is 32°C (27). The ELB agent has genotypic and phenotypic characteristics typical of the spotted fever group rickettsiae, and our failure to isolate the organism at human body temperature indicates that this relatively high temperature is not optimal for the efficient recovery of the organism, as is observed with other human pathogens (e.g., *Mycobacterium leprae*, *Yersinia* sp.). Our demonstration of the ability of XTC-2 cells to support the growth of the ELB agent indicates that this cell type is an efficient tool to test the growth of other *Rickettsia* species at lower temperatures. This cell line has also proven to be a versatile host for Bunyaviridae, including Bunyaviruses, α-viruses, flaviviruses, and rhabdoviruses (28).

The use of XTC-2 cells proved effective in recovering the ELB agent from fleas, with isolations from 19 of 20 macerated flea samples positive by PCR for the organism. Other spotted fever group rickettsia or arthropod-borne bacteria, such as *Wolbachia* sp. (29) and *Bartonella bacilliformis* (30), may also be cultivated more effectively at lower temperatures by using this cell line.

A pathogenic role of the ELB agent in four patients from Texas and Mexico has been demonstrated by PCR (6,31). However, because serologic tools for the organism were not available, the prevalence of infections by the ELB agent in different areas has yet to be determined. The ELB agent has been found in several species of fleas in the United States, including *C. felis* and *Pulex irritans* (5). These fleas, however, are prevalent worldwide, and we have detected DNA sequences of the ELB agent in Ethiopian fleas independently tested for another purpose. Although we did not obtain Brazilian fleas, we suspect that the ELB agent has a worldwide distribution in fleas.

Human infections with the agent also appear to be widespread, with our results showing that 2 French patients with clinical rickettsial disease and 2 of 16 Brazilian patients with febrile rash had high antibody titers to the ELB agent. Moreover, we confirmed that our PCR serology consistently identified specific sequences of the ELB agent in the serum of one patient; four such cases have been reported (6,30). Our findings indicate that further specific studies are required to determine the distribution of the ELB agent and the prevalence of the agent and associated infection, but this is the first report from cases outside the United States and Mexico.

The characteristics of our reference strain of the ELB agent, Marseille, differ phenotypically from those reported for the lost *R. felis* strain (6). Our strain does not grow at 35°C or 37°C, and its SDS–PAGE protein profiles and Western blots differed from those reported for *R. felis* and *R. typhi* (3,7,8). Further studies will determine if the reactive 30-kDa heat-labile protein observed on Western blot could be a truncated r*Omp*A protein, as predicted by genomic studies (31). By PCR and sequencing, we identified our isolates as the ELB agent on the basis of 100% *glt*A sequence homology with the sequence available for *R. felis* (5,6). Therefore, discrepancies with previously reported phenotypic findings may result from contamination of *R. felis* cultures with *R. typhi,* which was reported after experiments by the group that described *R. felis* (9). In our experiments based on PCR techniques, however, we found no evidence of contamination of our isolate with *R. typhi*. To avoid confusion between the characteristics of our isolate and those of the previously characterized *R. felis*, we are preparing a formal taxonomic characterization of our isolate of the ELB agent.

In summary, our experiments have demonstrated the usefulness of XTC-2 cells in isolating arthropod-associated microorganisms. This cell culture system allowed us to establish and make available the Marseille strain of the ELB agent. In addition, we have identified likely cases of infection by the ELB agent. The techniques that we describe should facilitate further studies to determine the prevalence and clinical spectrum of infection by this organism in humans.

### Acknowledgments

The authors thank J. Georgi of the El Labs in Soquel, California, for providing cat fleas, S.B. Calic and C.B. Chamone for providing Brazilian patients' serum specimens, and P.J. Kelly for review of the translation.

Dr. Raoult is Director of the Unité des Rickettsies, the national reference center for rickettsiosis and WHO collaborative center. The laboratory is mostly involved in the study of emerging and reemerging bacteria and arthropod-borne diseases.

### References

1. Raoult D, Roux V. Rickettsioses as paradigms of new or emerging infectious diseases. Clin Microbiol Rev 1997;10:694-719.
2. Adams JR, Schmidtmann ET, Azad AF. Infection of colonized cat fleas, *Ctenocephalides felis* (Bouché), with a rickettsia-like microorganism. Am J Trop Med Hyg 1990;43:400-9.
3. Higgins JA, Radulovic S, Schriefer ME, Azad AF. *Rickettsia felis:* a new species of pathogenic rickettsia isolated from cat fleas. J Clin Microbiol 1996;34:671-4.
4. Zavala-Velasquez JE, Sosa-Ruiz JA, Zavala-Castro J, Jimenez-Delgadillo B, Vado-Solis IE, Sanchez-Elias RA, et al. *Rickettsia felis*: the etiologic agent of three cases of rickettsiosis in Yucatan. Lancet 2000;356:1079-80.
5. Azad AF, Sacci JB, Nelson WM, Dasch GA, Schmidtmann ET, Carl M. Genetic characterization and transovarial transmission of a typhus-like rickettsia found in cat fleas. Proc Natl Acad Sci U S A 1992;89:43-6.
6. Schriefer ME, Sacci JB Jr, Dumler JS, Bullen MG, Azad AF. Identification of a novel rickettsial infection in a patient diagnosed with murine typhus. J Clin Microbiol 1994;32:949-54.
7. Azad AF, Radulovic S, Higgins JA, Noden BH, Troyer JM. Flea-borne rickettsioses: ecologic considerations. Emerg Infect Dis 1997;3:319-27.
8. Radulovic S, Higgins JA, Jaworski DC, Dasch GA, Azad AF. Isolation, cultivation, and partial characterization of the ELB agent associated with cat fleas. Infect Immun 1995;63:4826-9.
9. Radulovic S, Higgins JA, Jaworski DC, Azad AF. In vitro and in vivo antibiotic susceptibilities of ELB rickettsiae. Antimicrob Agents Chemother 1995;39:2564-6.
10. Trilar T, Radulovic S, Walker DH. Identification of a natural cycle involving *Rickettsia typhi* infection of *Monopsyllus sciurorum sciurorum* fleas from the nests of the fat dormouse (*Glis glis*). Eur J Epidemiol 1994;10:757-62.
11. La Scola B, Raoult D. Diagnosis of Mediterranean spotted fever by cultivation of *Rickettsia conorii* from blood and skin samples using the centrifugation-shell vial technique and by detection of *R. conorii* in circulating endothelial cells: a 6-year follow-up. J Clin Microbiol 1996;34:2722-7.
12. Pudney M, Varma MG, Leake CJ. Establishment of a cell line (XTC-2) from the South African clawed toad, *Xenopus laevis*. Experientia 1973;29:466-7.
13. Gimenez DF. Staining rickettsiae in yolk-sac cultures. Stain Technol 1964;39:135-40.
14. Teysseire N, Chiche-Portiche C, Raoult D. Intracellular movements of *Rickettsia conorii* and *R. typhi* based on actin polymerization. Res Microbiol 1992;143:821-9.
15. Eremeeva ME, Balayeva NM, Ignatovich VF, Raoult D. Proteinic and genomic identification of spotted fever group rickettsiae isolated in the former USSR. J Clin Microbiol 1993;31:2625-33.
16. Teysseire N, Raoult D. Comparison of Western immunoblotting and microimmunofluorescence for diagnosis of Mediterranean spotted fever. J Clin Microbiol 1992;30:455-60.
17. Laemmli UK. Cleavage of structural proteins during the assembly of the head of bacteriophage T4. Nature 1970;227:680-5.
18. Roux V, Fournier PE, Raoult D. Differentiation of spotted fever group rickettsiae by sequencing and analysis of restriction fragment length polymorphism of PCR amplified DNA of the gene encoding the protein rOmpA. J Clin Microbiol 1996;34:2058-65.

19. Roux V. Phylogenetic analysis and taxonomic relationships among the genus *Rickettsia*. In: Raoult D, Brouqui P, editors. Rickettsiae and rickettsial diseases at the turn of the third millennium. Marseille: Elsevier;1999. p. 52-66.
20. Dessen P, Fondrat C, Valencien C, Munier G. BISANCE: a French service for access to biomolecular databases. Cabios 1990;6:355-6.
21. Felsenstein J. PHYLIP-phylogeny inference package (version 3.2). Cladistics 1989;5:164-6.
22. Jukes TH, Cantor CR. Mammalian protein metabolism. In: Munro HN, editors. Evolution of protein molecules. New York: Academic Press; 1969. p. 121-32.
23. Saitou N, Nei M. The neighbor-joining method: a new method for sequences. J Mol Biol 1987;16:111-20.
24. Brown JKM. Bootstrap hypothesis tests for evolutionary trees and other dendograms. Proc Natl Acad Sci U S A 1994;91:12293-7.
25. Maurin M, Birtles RJ, Raoult D. Current knowledge of *Bartonella* species. Eur J Clin Microbiol Infect Dis 1997;16:487-506.
26. Perry RD, Fetherston JD. *Yersinia pestis:* etiologic agent of plague. Clin Microbiol Rev 1997;10:35-66.
27. Weiss E, Moulder JW. The Rickettsias and Chlamydias. In: Kreig NR, Holt JG, editors. Bergey's manual of systematic bacteriology. Baltimore: Williams & Wilkins; 1984; p. 687-739.
28. Watret GE, Pringle CR, Elliott RM. Synthesis of Bunyavirus-specific proteins in a continuous cell line (XTC-2) derived from *Xenopus laevis*. J Gen Virol 1985;66:473-82.
29. O'Neill SL, Pettigrew MM, Sinkins SP, Braig HR, Andreadis TG, Tesh RB. In vitro cultivation of *Wolbachia pipientis* in an *Aedes albopictus* cell line. Insect Mol Biol 1997;6:33-9.
30. Bouyer DH, Crocquet-Valdes PA, Walker DH. Expression and size determination of the rOmpa protein of *Rickettsia felis*. Proceedings of the 15th meeting of the American Society for Rickettsiology. Florida: ASR; 2000. p. 61.
31. Roux V, Rydkina E, Eremeeva M, Raoult D. Citrate synthase gene comparison: a new tool for phylogenetic analysis, and its application for the rickettsiae. Int J Syst Bacteriol 1997;47:252-61.
